# Bovine tuberculosis visible lesions in cattle culled during herd breakdowns: the effects of individual characteristics, trade movement and co-infection

**DOI:** 10.1186/s12917-017-1321-z

**Published:** 2017-12-29

**Authors:** Andrew W. Byrne, Jordon Graham, Craig Brown, Aoibheann Donaghy, Maria Guelbenzu-Gonzalo, Jim McNair, Robin Skuce, Adrian Allen, Stanley McDowell

**Affiliations:** 1 Agri-food and Biosciences Institute, Veterinary Science Division, Stormont, Belfast, Northern Ireland BT43SD UK; 2 School of Biological Sciences, Queen’s University Belfast, Belfast, Northern Ireland UK

**Keywords:** Bovine TB, *Mycobacterium bovis*, BVDV, Liver fluke, Paratuberculosis, Co-infection, Johne’s disease, MAP, Fascioliasis, Gamma interferon, Skin tests, Cattle

## Abstract

**Background:**

Bovine tuberculosis (bTB), caused by *Mycobacterium bovis*, remains a significant problem for livestock industries in many countries worldwide including Northern Ireland, where a test and slaughter regime has utilised the Single Intradermal Comparative Cervical Tuberculin (SICCT) test since 1959.

We investigated the variation in post-mortem confirmation based on bTB visible lesion (VL) presence during herd breakdowns using two model suites. We investigated animal-level characteristics, while controlling for herd-level factors and clustering. We were interested in potential impacts of concurrent infection, and therefore we assessed whether animals with evidence of liver fluke infection (*Fasciola hepatica*; post-mortem inspection), *M. avium* reactors (animals with negative *M. bovis-avium* (*b-a*) tuberculin reactions) or Bovine Viral Diarrhoea Virus (BVDV; RT-PCR tested) were associated with bTB confirmation.

**Results:**

The dataset included 6242 animals removed during the 14 month study period (2013–2015). bTB-VL presence was significantly increased in animals with greater *b-a* reaction size at the disclosing SICCT test (e.g. *b-a* = 5-9 mm vs. *b-a =* 0 mm, adjusted Odds ratio (aOR): 14.57; *p* < 0.001). *M. avium* reactor animals (*b-a* < 0) were also significantly more likely to disclose VL than non-reactor animals (*b-a =* 0; aOR: 2.29; *p* = 0.023). Animals had a greater probability of exhibiting lesions with the increasing number of herds it had resided within (movement; log-herds: aOR: 2.27–2.42; *p* < 0.001), if it had an inconclusive penultimate test result (aOR: 2.84–3.89; *p* < 0.001), and with increasing time between tests (log-time; aOR: 1.23; *p* = 0.003). Animals were less likely to have VL if they were a dairy breed (aOR: 0.79; *p* = 0.015) or in an older age-class (e.g. age-quartile 2 vs. 4; aOR: 0.65; *p* < 0.001). Liver fluke or BVDV variables were not retained in either multivariable model as they were non-significantly associated with bTB-VL status (*p* > 0.1).

**Conclusions:**

Our results suggest that neither co-infection of liver fluke nor BVDV had a significant effect on the presence of VLs in this high-risk cohort. *M. avium* tuberculin reactors had a significantly increased risk of disclosing with a bTB lesion, which could be related to the impact of co-infection with *M. avium* subsp. *paratuberculosis* (MAP) affecting the performance of the SICCT however further research in this area is required. Movements, test history, breed and age were important factors influencing confirmation in high-risk animals.

**Electronic supplementary material:**

The online version of this article (10.1186/s12917-017-1321-z) contains supplementary material, which is available to authorized users.

## Background

Despite decades of research into bovine tuberculosis (bTB), caused by *Mycobacterium bovis*, there still remains limitations in our ability to explain the variation in bTB detectability and confirmation infection within cattle [1–4]. Furthermore, animal-level analyses relating to pathological findings from surveillance have not been as frequently investigated, in comparison with epidemiological studies concentrating on herd-level factors [5, 6].

Bovine TB in Northern Ireland remains a significant chronic problem, with continual maintenance of the disease in the national herd despite a coordinated compulsory statutory scheme in place since the 1950s [7, 8]. This national scheme has been predicated on an ante-mortem test and cull regime, and supplemented with additional continual slaughter house surveillance systems [7]. The Single Intradermal Comparative Cervical Tuberculin (SICCT) test is the statutory ante-mortem test, and as dictated by legislation, all animals deemed to be standard reactors are compulsorily culled. Additional animals may be culled if ‘severe’ interpretation of the test is implemented during herd breakdowns, or through the ancillary use of the Interferon-γ test or serological tests, in an attempt to remove exposed animals [9–11]. However, all of these tests have limitations in terms of sensitivity (especially SICCT and serological tests) and specificity (especially the interferon-γ test), meaning some infected animals can be missed from breakdown herds or some non-infected animals needlessly culled [9, 12–14].

Recently, co-infection of other pathogens has been highlighted as a potential factor affecting the efficacy of the current systems to identify and remove infected animals from endemic bTB countries like the UK and Ireland [15–22]. Bovine Viral Diarrhoea Virus (BVDV) [15], liver fluke (*Fasciola hepatica*; [19]) and Johne’s disease (caused by *Mycobacterium avium* subsp. *paratuberculosis* [21, 23]) have all been suggested to be implicated in the epidemiology of bovine TB either by actively exacerbating infection, potentially leading to increase *M. bovis* transmission, increasing host susceptibility, or by affecting the performance of tests used to detect bTB infection.

Here we investigate the variation in post-mortem confirmation (presence of bTB visible lesions; VL) of at-risk animals culled in Northern Ireland during 2013–2015. All animals removed were ante-mortem positive for bTB, or negative at-risk animals in direct contact with infected animals. Building on the previous work by O’Hagan et al. [4] in Northern Ireland, we hypothesised that confirmation would be significantly associated with variations in the SICCT test reaction size, the animal’s test history, movement history (trade), and herd-level characteristics where the animal resided before slaughter [1, 3, 4]. Controlling for such risk factors, we then also utilised these data to explore the hypotheses that co-infection (exposure to BVDV, liver fluke or non-bTB *Mycobacterium*) may be significantly associated (either in positively or negatively) with the probability of bTB confirmation.

## Methods

The study design was a retrospective observational case-control study. The study population was animals slaughtered as part of bTB herd breakdowns (where bTB has been detected in the herd via one or more positive statutory tests) in Northern Ireland. Cases were exposed animals with bTB visible lesions (VL), a metric of post-mortem confirmation, as used elsewhere (e.g. [1–4, 24–27]). Controls were animals without VL detected at slaughter. 97% of animals with lesions were laboratory confirmed (histopathology and/or culture) and a previous risk factor study of bovine reactors from Northern Ireland suggested that little difference in parameter estimates occurred when comparing VL with bacteriologically confirmed samples [4]. Extensive details of the TB program in Northern Ireland are presented in [7], and specific details of the processing of reactors and the determination of cases is presented in [4]. Briefly, reactor animals are sent to slaughter where post-mortem examination (PME) within the abattoir for evidence of infection through the presence of VLs is undertaken [4]. During the study period, 99% of samples were processed in one specific slaughterhouse, which would reduce the inter-abattoir variability in detection of lesions [1, 2]. The routine PME includes palpation of the lungs, liver and tongue, and incision of the lungs, heart, and liver. In reactor animals, examination of the lymph nodes of the head, chest and mesenterium, lungs, pleura, peritoneum, and the prescapular, popliteal, iliac, precrural lymph nodes was undertaken [4].

The dataset contained records from animals that were culled from the 27th of November 2013 to the 27th of January 2015. All animals were culled as they were deemed reactors to the Single Intradermal Comparative Cervical Tuberculin (SICCT) test (either standard interpretation or severe interpretation), gamma interferon test positive or animals in-contact with test positive animals.

### Metrics of co-infection

The primary goal of this study was to investigate potential interactions between endemic diseases and *M. bovis* confirmation (VL). We investigated whether there was any evidence of interaction between BVDV positivity or exposure, liver fluke post-mortem status, and *Mycobacterium avium* complex exposure. All culled animals in the dataset had a tissue sample (ear-tag notch) available, which was stored at −70°c. These samples were used to prospectively test for BVDV virus using a commercial real time RT-PCR test (Virotype BVDV RT-PCR, Qiagen) as described before [22, 28]. All samples were first batch screened by RT-PCR, and individual samples within positive batches were then tested using antigen-capture ELISA test (Herd Check BVDV antigen/serum plus, IDEXX Laboratories) to detect viraemic animals. BVDV-bTB interaction was then assessed using two variables: (i) a direct BVDV RT-PCR positive test assigned to the animal, indicating the animal has active infection with BVDV (ii) indirect herd-level exposure, with all animals within a herd with a positive BVDV animal being considered exposed. Liver fluke status was also assessed with three metrics of infection using surveillance data [29]: (i) *Fascioliasis* recorded at slaughter, indicating that active infection (the presence of *Fasciola hepatica* in the liver) was occurring at time of death (1 = active infection; 0 = non-active infection) (ii) Liver fluke damage recorded at slaughter, indicating that the animal had been infected in the past, with resulting scar tissue in the liver, however the animal was not currently infected (1 = historic infection; 0 = non-historic infection) (iii) the animal was either found to have *Fascioliasis* or liver fluke damage at slaughter (1 = active infection or historic infection; 0 = non-active and non-historic infection). Please note, that category three is related to the other categories and so could not be entered into the same model.

Potential exposure to non-bTB *Mycobacterium* was assessed using the relative reaction size to the bovine and *M. avium* Purified Protein Derivative (PPD) tuberculin used during the disclosing test [30–33]. The comparative skin test (SICTT) used in Northern Ireland assigns test status to animals based on the bovine tuberculin reaction size subtracted by the avian reaction size (*b-a*), with positive bias values indicating a reaction to the bovine tuberculin greater than to the avian tuberculin (i.e. net bias), and negative reaction sizes indicating a reaction to avian tuberculin greater than to the bovine tuberculin. A bTB “standard reactor”, one deemed positive to the statutory test and required to be culled by law, is one whereby the *b-a* > 4 mm. The relationship between PPD reaction size and VL presence was non-linear (Additional file 1; and see Additional file 2) – and was modelled by categorising the values into one of six types, three non-standard reactors (1. Avium reactor (*b-a* < 0 mm), 2. Non-reactor (*b-a* = 0 mm); 3. Inconclusive bovine reactor (*b-a* > 0 & < =4 mm)) and three standard reactor categories (4. “Small bovine reactors” (*b-a* > 4 & *b-a* < 10 mm); 5. “Large bovine reactors” (*b-a* > =10 & *b-a* < 20 mm); 6. “Hyper bovine reactors” (*b-a* > =20 mm)). We considered *M. avium* reactors as potentially indicative of exposure to non-bTB *Mycobacterium,* including *Mycobacterium avium* subsp. *paratuberculosis* the agent of Johne’s disease in cattle [30, 32, 33], a notifiable pathogen in Northern Ireland. To further explore the impact of *M. avium* reactors on lesion presence, the effect of the individual bovine PPD reaction was assessed using descriptive statistics and univariable models. For this, a single intradermal tuberculin (SIT) test results was established as positive if the net bovine PPD reaction was >4 mm.

### Other co-factors

We concentrated on animal-level variables in this study, while controlling for herd-level clustering. Factors investigated in our models are presented Table 1. These variables were chosen as we hypothesised that they could be influential in the ante-mortem test variability, confirmation, or pathology or because previous research suggested that they may be important (e.g. [3–5, 16, 25]).

**Table 1:** Variables assessed for association with post-mortem presence of bTB visible lesions

Variable	Description
Reason for removal	Categorical; 1. Negative in contact; 2. IFN-γ; 3. SICCT
Bovine reaction size	Reaction to bovine tuberculin injection (in mm) during skin testing for bTB
Avian reaction size	Reaction to avian tuberculin injection (in mm) during skin testing for bTB
*b-a* reaction difference	Bovine reaction minus avian reaction to tuberculin injection during skin testing for bTB (in mm)
BVD test result	Binary; RT-PCR test result for ear-tag samples
Inconclusive BVDV result	Binary; inconclusive RT-PCR test result for ear-tag samples
Indirect BVDV test result	Binary; if one animal in a herd tests + for BVDV then all animals from that herd gets assigned an indirect +; otherwise -
Fascioliasis	Binary; Presence of active infection of *F. hepatica* = 1; absence of active infection = 0
Fluke damage	Binary; Presence of fluke damage = 1; absence of fluke damage = 0
Fluke general	Binary; Presence of either Fascioliasis or fluke damage = 1; absence of Fascioliasis and absence of fluke damage = 0
VL	Binary; presence of a bTB like visible lesion (VL)
Age-quartiles	Animal age in years at death; categorised into quartiles.
Breed	Breed of animal (breeds associated with dairy or non-dairy)
Sex	Male or female
Associated herds	Number of herds from birth to death
Number of lifetime moves	Number of movements from birth to death, including market moves
Time since last test	Time from penultimate to ultimate test
bTB Herd history (4 years)	Binary, whether the last herd the animal resided in had a history of bTB over the preceding 4 years
Penultimate SICCT reading	Whether the animal was a severe interpretation reaction at penultimate test (binary)
Total reactors at disclosure	Total number of reactors disclosed during the breakdown where the animal was SICCT positive (Breakdown size). Note, that the breakdown was defined as the total period over which the herd was restricted, with the total number animals removed over that period.
Herd size	Total animals within the herd at time of testing; categorised into quartiles
Number of purchased animals	Number of bought-in animals during the last 3 years; categorised into quartiles
Region	Location based on three categories (North, South-west, South-east)

### Response variable and modelling approach

We developed models where the outcome was binary - bTB VL presence or absence [1–4]. The primary source of data for these analyses was the Northern Ireland Animal and Public Health Information System (APHIS; [34]). The presence of a lesion at slaughter was modelled using a multivariable binary logit random effects model, with the random effect capturing the potential non-independence of animals coming from the same herds [35]. The significance of this clustering was measured using a likelihood ratio test, compared with a regular logit regression model. Unlike previous studies [4], we did not include slaughter house as a random effect, as 99% of all animals within the dataset were processed at the same abattoir.

Throughout we used a two-step model building strategy – univariable model screening and multivariable model building. The functional form was assessed using LOWESS graphing, with suitable transforms of predictors implemented to improve model fit (e.g. natural logarithm transform; Additional file 1; Additional file 3; and see Additional file 2). We fitted separate univariable models for each independent variable of interest, and all variables that were significant at *p* < 0.15 were candidates for a multivariable model building process. The variables tested during the model building for the logistic regression model are presented in Table 1 below. Correlations amongst independent variables were assessed, and where necessary, variables were dropped if highly correlated (i.e. to reduce the possibility of multi-collinearity; [35, 36]). The decision to include a variable amongst a correlated pair was based on which variable improved the model fit more, as assessed by Akaike’s Information Criteria (AIC). Backwards elimination was then used to establish parsimonious models [35], with final competing models compared using AIC and Bayesian Information Criteria (BIC) values [37, 38]. Models with lower Information Criteria (IC) values were considered preferred models. It should be noted, that ICs could not be used to compare models of differing number of observations [37]. In that case (for example, model with or without the *b-a* variable), were considered simply as separate competing models, and duly reported. First order interaction terms were fitted for variables retained after backwards model building, and only retained if they improved the fit of the model and were significant at an *p* < 0.05. Covariate patterns were assessed using visual plots. The discriminatory ability of the final models were assessed by estimating the Area Under the ROC Curve (AUC). Models with an AUC > 0.7 were considered “adequate” [39]. All modelling was undertaken with Stata SE 14 (Stata Corp., 2015).

## Results

### Descriptive results of the dataset

The dataset included a total of 6242 animals with an ante-mortem and post-mortem record, from 1335 herds; however not all variables in the dataset were complete. Overall, 5198 animals were recorded in the dataset as “positive” skin reactors (5198/6242; 83%). A further 458 animals (458/6242; 7.3%) were designated as gamma positive, with 586 (586/6242; 9.4%) being designated as negative in contacts (NIC). Of these 6242 animals with an associated post-mortem result, 40% (2504) had visible lesions disclosed at slaughter. Of the 5198 standard reactor animals within the dataset, 2444 (47%) disclosed with visible lesions at slaughter.

Overall, there were 5698 animals with a *b-a* value recorded at the disclosing SICCT test (Table 2). Descriptive statistics relating to the breakdown and VL percentage are presented in Table 2. Not all animals were tested for gamma interferon, as this test was a supplementary test to the SICCT tests. There were 355 of these animals with gamma interferon as their disclosing test (355/5698; 6.2%), 20 of which were VL positive (5.6%). There were 20 animals from 5698 that were negative to all ante-mortem tests (20/5698; 0.35%); 2 were VL positive (10%). Five hundred ninety-six animals were inconclusive reactors to both standard and severe interpretation SICCT tests (596/5698; 10%), 142 of which were VL positive (24%). There were 259 animals that were inconclusive to severe interpretation but negative to both gamma and standard (259/5698; 4.6%; Table 2), 19 of which were VL positive (7.3%).

**Table 2:** Tabulation of bTB test results by standard interpretation (positive, negative or inconclusive) SICCT, severe (sev.) interpretation SICCT, and Interferon-γ, and the percentage (%) which disclosed bTB visible lesions (VL) at slaughter

	Gamma negative/untested (% VL+)			Gamma positive (% VL+)			Row totals
Standard interpretation	Sev. Neg.	Sev. Pos.	Sev. Incon.	Sev. Neg.	Sev. Pos.	Sev. Incon.	
Neg.	2/20 (10%)	0	19/259 (7%)	3/20 (15%)	0	16/320 (5%)	40/619 (6%)
Pos.	0	2224/4235 (53%)	0	0	0	0	2224/4235 (53%)
Inconclusive	0	69/248 (28%)	138/581 (24%)	0	0	4/15 (27%)	211/844 (21%)
Column totals	2/20 (10%)	2293/4483 (51%)	157/840 (19%)	3/20 (15%)	0	20/335 (6%)	

The gamma results only relate to animals designated for removal due to the gamma test result only; these data only relate to the cohort of animals where a *b-a* reading was recorded (*n* = 5698)

### Factors associated with lesion presence - *Univariable models*

We present the univariable associations in Table 3, however a thorough description of the univariable model findings is presented in Additional file 2.

**Table 3:** Univariable random effects logit models predicting the probability of an animal disclosing with a visible lesion at slaughter

Variable (outcome = VL)	N-positive (% positive)		N	OR (95%CI)	*P*-value
Risk factors at animal level					
*b-a* PPD reaction size			5698		<0.001
	18 (11.92%)	<0 mm		2.39 (1.19–4.82)	
	22 (4.7%)	0 mm		Ref.	
	211 (25%)	1-4 mm		7.16 (4.32–11.87)	
	691 (36.64%)	5-9 mm		15.97 (9.8–26.01)	
	884 (59.81%)	10-19 mm		45.47 (27.8–74.37)	
	649 (74.51%)	>20 mm		109.53 (65.52–183.10)	
Reason for removal			6242		<0.001
	30 (5.12%)	Negative in contact		Ref.	
	30 (6.55%)	Removed due to IFN-γ result		1.32 (0.72–2.38)	
	2444 (47.02%)	Removed due to SICCT result		20.83 (13.58–31.95)	
BVD			6242		0.106
	2500 (99.84%)	BVD Negative		Ref.	
	4 (0.16%)	BVD positive		0.33 (0.08–1.26)	
BVD (indirect)			6242		0.165
	2407 (40.41%)	BVD Negative		Ref.	
	97 (33.92%)	BVD positive		0.60 (0.29–1.23)	
Fascioliasis			6242		0.292
	2372 (40.33%)	Fascioliasis Negative		Ref.	
	132 (36.57%)	Fascioliasis Positive		0.86 (0.65–1.13)	
Liver fluke damage			6242		0.997
	2161 (40.27%)	Liver damage Negative		Ref.	
	343 (39.16%)	Liver damage Positive		1.00 (0.83–1.2)	
Liver fluke exposure			6242		0.55
	2029 (40.53%)	Facioliasis and damage Negative		Ref.	
	475 (38.43%)	Facioliasis or damage Positive Mean		0.95 (0.81–1.11)	
Age-quartiles			6231		<0.001
	612 (39.74%)	≤ 1.53 years		0.77 (0.64–0.92)	
	704 (45.48%)	1.53–2.86 years		Ref.	
	580 (36.64%)	2.86–5.58 years		0.69 (0.58–0.83)	
	607 (38.91%)	≥5.58 years		0.76 (0.63–0.91)	
Breed			6242		<0.001
	1517 (44.29%)	Non-dairy		Ref.	
	987 (35.04%)	Dairy		0.73 (0.61–0.86)	
Sex			6242		0.406
	517 (39.26%)	Male		Ref.	
	1987 (40.35%)	Female		1.07 (0.90–1.26)	
Associated herds			6242		<0.001
		Log(No. associated herds)		2.84 (2.45–3.30)	
No. Market moves			6242		0.028
		Market moves		1.04 (1–1.08)	
Time since last test			5831		<0.001
		Log(Time in days between tests)		1.59 (1.40–1.80)	
Herd bTB history			6242		<0.001
	1188 (35.83%)	No bTB		Ref.	
	1316 (44.98%)	bTB		2.13 (1.76–2.57)	
Penultimate herd bTB history			6242		0.044
		No bTB		Ref.	
		bTB		2.02 (1.02–4.00)	
Penultimate test results			6242		<0.001
	2246 (39.94%)	Severe interpretation negative on penultimate test		Ref.	
	84 (59.15%)	Severe interpretation positive on penultimate test		2.42 (1.57–3.72)	
	174 (36.48%)	No previous test result		0.84 (0.64–1.09)	
Breakdown size			6242		0.001
		per additional reactor		0.99 (0.98–0.99)	
Herd size			6237		0.044
(quartiles)	716 (45.99%)	≤71		Ref.	
	637 (41.26%)	72–158		0.86 (0.66–1.1)	
	491 (33.22%)	159–279		0.71 (0.54–0.93)	
	658 (39.69%)	≥280		0.72 (0.54–0.94)	
Purchases			6017		0.114
(quartiles)	593 (41.61%)	≤7			Ref.
	683 (43.98%)	8–31		1.12 (0.86–1.46)	
	542 (35.38%)	32–100		1.22 (0.91–1.64)	
	582 (38.62%)	≥101		1.44 (1.06–1.94)	
Region			6242		0.171
	629 (46.35%)	1. North (Derry, Larne, Ballymena and Coleraine)		Ref.	
	863 (38.2%)	2. South-West (Omagh, Enniskillen and Dungannon)		0.91 (0.7–1.18)	
	1012 (38.54%)	3. South-east (Newtownards, Newry and Armagh)		0.78 (0.61–1.01)	

### Multivariable random effect logit model – Lesion presence

After univariable model fitting, there were 14 independent variables offered to the full model (*p* < 0.15 in univariable models). Correlation analysis amongst independent variables found that the lifetime number of market movements and the number of herds (log(no. associated herds)) the animal resided in during its lifetime were strongly correlated (*r* = 0.83; *p* < 0.001). We retained only log(no. associated herds) variable to avoid collinearity in our model, and because this was a far superior predictor of lesion presence in the univariable model (ΔAIC: 208). Purchase also significantly correlated with log(no. associated herds) (*r* = 0.46; *p* < 0.001), but was not significantly associated with the outcome at univariable level (*p* = 0.114) and so was also dropped from the model building process.

Two competing models were run before model building with either the *b-a* reaction size included or the test result (skin test positive, gamma positive, or NIC animals). This was due to the number of animals without a *b-a* value (*n* = 544 missing values); 81% (440/544) of these animals were NIC animals.

The final model (Table 4) including *b-a* as a predictor significantly explained variation in the dataset (Wald χ^2^ (df: 9) = 772.86; *p* < 0.001), and displayed significant variation in animal risk across herds (σ: 1.13; ρ: 0.28; LR test of ρ = 0: *p* < 0.001). The model exhibited adequate discriminatory ability with an AUC of 0.75 (95%CI: 0.74–0.77). The model was based on 5698 observations, with an average of 4.3 animals per herd. The probability of an animal disclosing a lesion at slaughter varied significantly by *b-a* reaction size, the number of herds the animal resided in during its lifetime, breed and penultimate test result. Animals with net positive bovine increase (*b-a* > 0) had a significantly higher risk of having a lesion at slaughter (*P* < 0.001), than non-reactors. Furthermore, this pattern increased with increasing *b-a* size; for example, in comparison with non-reactors (*b-a* = 0), animals with reaction sizes of 1-4 mm were 6.31 times more likely to have a lesion (*p* < 0.001), whereas animals with reaction sizes 5-9 mm were 14.57 times more likely to have a lesion (*p* < 0.001). Animals who were *M. avium* tuberculin reactors (*b-a* < 0; *n* = 151) were also significantly more likely to disclose with a lesion relative to non-reactor animals (OR: 2.29; *p* = 0.023).

**Table 4:** Random effect multivariable logit model assessing the variation in the presence of bTB visible lesions. Test result modelled as categories of the bovine-avian Purified Protein Derivative (PPD) tuberculin reaction size (*n* = 5698)

Predictor	Categories	OR	Std. Err.	z	*P*	Lower 95% CI	Upper 95% CI
*b-a PPD reaction size*							
	<0 mm	2.29	0.83	2.28	0.023	1.12	4.65
	0 mm	Ref.					
	1-4 mm	6.31	1.64	7.07	<0.001	3.79	10.51
	5-9 mm	14.57	3.66	10.66	<0.001	8.90	23.85
	10-19 mm	42.59	10.79	14.81	<0.001	25.92	69.98
	>20 mm	103.03	27.29	17.50	<0.001	61.31	173.15
Log(No. associated herds)		2.27	0.19	9.76	<0.001	1.92	2.67
Breed	Non-dairy	Ref.					
	Dairy	0.79	0.08	−2.43	0.015	0.65	0.96
Penultimate test results	Negative	Ref.					
	Severe interpretation positive	3.98	0.93	5.93	<0.001	2.52	6.28
	No previous test result	1.08	0.17	0.48	0.629	0.79	1.48
Constant		0.02	0.01	−14.95	<0.001	0.01	0.03
RE	ρ^a^	0.28	0.03			0.23	0.33

^a^Intra-class correlation

There was significant positive relationship between the log-number of herds an animal resided within and lesion risk (log-herds: OR: 2.27; *p* < 0.001). Dairy breed animals had significantly lower odds of having a lesion at slaughter relative to non-dairy animals (OR: 0.79; *P* = 0.015). Animals that were positive reactors on their penultimate skin test under severe interpretation were 3.98 times more likely to have a lesion at slaughter than negative animals. There was no significant difference between animals without a penultimate test result and those who tested negative (*p* = 0.201).

The second model significantly explained variation in the dataset (Wald χ^2^ (df: 9) = 448.18; *p* < 0.001; Table 5), and revealed significant variation in the probability of lesion disclosure across herds (significant herd random effect; LR test of ρ = 0: *p* < 0.001). The model exhibited fair to adequate discriminatory ability with an AUC of 0.69 (95%CI: 0.67–0.70). The final model contained 5823 animals, from 1286 herds, with an average of 4.5 animals per herd. Animals were significantly more likely to disclose with lesions if they were removed due to being SICCT test reactors (OR: 14.62; *p* < 0.001) in comparison with negative in-contact animals. There was no significant difference in the probability of VL presence between negative in-contacts and animals removed due to gamma interferon results (*p* = 0.738). Animals with a positive penultimate severe interpretation result were significantly more likely to have a lesion than (severe interpretation) test negative animals (OR = 2.84; *p* < 0.001). There was no difference between animals without a penultimate test result and test negative animals (*p* = 0.135). There was significant variation in the probability of a lesion across age quartiles; the highest risk was associated with quartiles 1 and 2. Animals in age quartile 3 (OR: 0.63) and quartile 4 (OR: 0.65) were of significantly lower odds of having a lesion relative to quartile 2 (*P* < 0.001), respectively. There was no significant difference between age quartile 1 and 2 (*p* = 0.446). There was a significant higher odds of lesion detection with the increasing (log) number of herds an animal resided within during their life (OR per unit increase: 2.42; *p* < 0.001). Finally, there was a significant positive relationship between lesion presence and time since last (penultimate) test (OR per log unit increase in days: 1.23; *p* = 0.003).

**Table 5:** Random effect multivariable logit model assessing the variation in the presence of bTB visible lesions. Bovine TB test result modelled as categories (reasons) leading to animal removal (*n* = 5823)

Predictor	Categories	OR	Std. Err.	z	*P*	Lower 95% CI	Upper 95% CI
Reason for removal	Negative in contact	Ref.					
	Removed due to IFN-γ result	1.11	0.34	0.33	0.738	0.61	2.03
	Removed due to SICCT result	14.62	3.32	11.80	<0.001	9.36	22.83
Penultimate test results	Negative	Ref.					
	Severe interpretation positive	2.84	0.65	4.54	<0.001	1.81	4.46
	No previous test result	0.62	0.20	−1.49	0.135	0.33	1.16
Age-class	≤ 1.53 years	1.08	0.11	0.76	0.446	0.88	1.33
	1.53–2.86 years	Ref.					
	2.86–5.58 years	0.63	0.06	−4.77	<0.001	0.53	0.76
	≥5.58 years	0.65	0.06	−4.37	<0.001	0.54	0.79
Log(No. associated herds)		2.42	0.19	11.26	<0.001	2.07	2.82
Log(Time in days between tests)		1.23	0.09	2.95	0.003	1.07	1.41
Constant		0.01	0.00	−11.15	<0.001	0.01	0.02
RE	ρ^a^	0.26	0.02			0.21	0.31

^a^Intra-class correlation

### Additional analysis of non-reactors and net *M. avium* PPD reactors

There were 151 animals with net increases in *M. avium* PPD reactions (*b-a < 0*), 37 of which would have been SIT reactors (24.5%). 11.92% (18/151) of these animals had VLs. 7/37 SIT positive animals had VLs (18.9%); 11/103 non-SIT animals had VLs (9.7%). This difference in the probability of lesion disclosure was non-significant (OR: 2.18; *p* = 0.136). The mean *M. avium* PPD reaction size for avium reactors with lesions was 5.82 mm (*n* = 11; SD: 3.12; Range: 2-12 mm), while the mean *M. avium* PPD reaction size for avium reactors without lesions was 4.01 mm (*n* = 103; SD: 2.05; Range: 1–12 mm), a significant difference (linear regression: *p* = 0.010).

For non-reactors (*b-a = 0*), 22/468 (4.7%) of animals had VLs. There was a significantly greater proportion of VL positive animals with positive SIT test results (16.0% (4/25)) than SIT negative animals (4.1%; 18/443; OR: 4.50; *p* = 0.011). For animals with a *b-a = 0* test reading, the mean *M. avium* PPD reaction size was non-significantly larger in animals with a VL (0.83 mm; range 0-4 mm) than animals without a VL (0.49 mm; range 0-4 mm) at slaughter (linear regression: *p* = 0.242).

## Discussion

Overall, this study has shown that there is significant variation in the probability of cattle exhibiting a VL at slaughter that were culled as part of herd breakdowns in Northern Ireland. This variation can be attributed to animal-level characteristics, exposure during life histories, and potentially to exposure to other *Mycobacteria*.

### Co-infection

Co-infections have been highlighted as a significant modulator of immune responses to the challenge of bacterial infections, including tuberculosis [40–44]. Recent studies suggest that bovine TB could be affected, either through mechanisms of increased pathology, increased transmission, susceptibility or decreased detection, by pathogens such as BVDV [15, 22, 45], liver fluke (*F. hepatica*; 18, 19, 44], and Johne’s Disease (JD; [20, 42, 46]; but see [47]).

During the present study BVDV exposure was assessed in two ways – a positive BVDV RT-PCR test result, or an indirect exposure metric based on the presence of a test positive animal disclosed within the animal’s final herd. The first metric represent Persistently Infected (PI) animals, which were rare in the dataset (0.27%), with the second metric representing potentially ‘exposed’ cattle. We did not find any significant (at *P* < 0.05) animal-level associations between these BVDV statuses and the probability of an animal having a lesion at slaughter. This could be partially due to the small number of test positive animals found within the cohort, reducing the power to detect an effect. Other research from this cohort suggested that the apparent indirect exposure of animals in herds where BVDV positive cattle were found was negatively associated with the minimum lesion counts found in animals – a finding which would not be generally consistent with the proposed mechanisms by which BVDV might impact bTB epidemiology [15, 22, 45, 48, 49]. Some have suggested that acute BVDV infection could lead to the rapid progression of bTB in co-infected animals [50]. However, experimental studies failed to show significant differences in *M. bovis* shedding patterns between co-infected (BVDV and *M. bovis*) and infected (*M. bovis* only) animals [15]. Previous research suggested that acute BVDV infection could temporarily compromise *ante-mortem* tests for bTB, by suppressing the immunological response to tuberculin PPDs [45]. Herd-level research from Northern Ireland suggested that, when controlling for confounders (especially herd-size which is a herd-level risk factor for both bTB and BVDV), there was no significant relationship between bTB and BVDV herd risk based on 2827 herds [22]. Results from populations outside Northern Ireland suggest that acute BVDV cases may in some cases exacerbate infection [50] or impact on *ante-mortem* tests ability to detect *M. bovis* in truly infected animals [45], but that these affects are not readily demonstrated with herd- and animal-level epidemiological population-level datasets in Northern Ireland [22]. However, as pointed out previously [22], biosecurity practices implemented in the management of BVDV could potentially have benefits for bTB management (but see [51]).

Liver fluke (*F. hepatica*) infection has been suggested to negatively impact the control of *M. bovis* in Britain and Ireland [18, 19, 44, 52]. Experimental work has found that a proportion of co-infected animals with Bacille Calmette Guerin (BCG) can be misdiagnosed using IFN-γ or SICCT tests [52]. Claridge et al. [19] presented both experimental and epidemiological evidence to suggest that co-infection with liver fluke was associated with reduced SICCT tests disclosure (underascertainment of 27–38%) in England and Wales. Experimental data presented in that paper suggested that co-infected animals exhibited significantly lower *b-a* reactions to the skin test at 10 and 21 weeks post-innoculation [19]. However, at both time points all co-infected animals would still have been deemed standard reactors (i.e. *b-a* > 4 mm). Despite these experimental and epidemiological data, during the present study, three metrics of liver fluke infection (Fascioliasis, fluke damage, and either Fascioliasis/fluke damage) were tested and none were significantly associated with the probability of an animal having a visible lesion at slaughter. However, other research from this population has suggested a negative relationship between maximum visible lesion size and liver fluke presence has been found [53]. This appears perhaps contrary to the hypothesis to an immunological suppression hypothesis [54–56], but reflects other recent data suggesting the potential for the induction of latency [57]. Further research is on-going into this latter hypothesis (Byrne et al. In prep.).

There was evidence from the present study that reaction to *M. avium* Purified Protein Derivative (PPD) tuberculin (negative *b-a* values), was significantly associated with visible lesion presence at slaughter. These animals would not have been classed as standard reactors to bovine tuberculosis, suggesting that animals with bTB visible lesions that strongly react to *M. avium* tuberculin will have increased risk of going undetected. The *M. avium* PPD comparator was introduced to increase specificity in areas where non-specific reactions could occur [13], however at a potential loss of sensitivity relative to the SIT test. For non-reactor animals and *M. avium* PPD reactors, a proportion of animals strongly reacted to the bovine PPD, and would have been classed as SIT reactors. It is possible that such *M. avium* reactors have been exposed to non-*bovis* mycobacteria, including *Mycobacterium avium* subsp. *paratuberculosis* (MAP) the etiological agent of Johne’s disease (JD; [30, 32, 33, 58]). Previous research has suggested that the comparative skin test can be used as a preliminary diagnostic for JD in cattle [32]. Gilardoni et al. [32] suggests animals with JD react positively to both bovine and avian tuberculins, but generally greater intensity to avian PPD. However, because of the non-specific nature of the avium tuberculin reaction, it is not possible to disaggregate exposure to MAP or other members of the *M. avium* complex. Recently in Ireland, Kennedy et al. [33] found a significant association between the reactions sizes (most markedly in animals reacting to both avian and bovine PPD) during a SICCT test and an ELISA test for MAP. Increases in tuberculin sizes at day 10 and day 16 post-inoculation was associated with increases in sample/positive ratio for the JD ELISA blood test. However, this study failed to elucidate whether tuberculin reaction sizes can be indicative of exposure to MAP, or if their results were due to cross reactivity. Our findings need to be further investigated with a larger dataset, and from cohorts that include non-breakdown herds, to verify whether this is a general pattern. Further research investigating specifically JD and bTB is on-going in Northern Ireland (Lahuerta-Marin, Byrne et al., personal communication). It should be noted that previous research has found significant effects of JD on bTB in cattle herds ([32, 42, 46, 59, 60], and vice-versa: [61]), and so co-management should be considered if managing both infections when present in the same herd, and careful attention to the interpretation of comparative tests where co-infection is suspected.

### Individual characteristics, herd-level factors, and life histories

Animals classed as of a dairy breed exhibited significant differences to beef breed types in post-mortem confirmation of bTB VL. Dairy breeds were dominated by Friesian and Holstein breeds (91% of total); in comparison, beef breeds were made up of a number of dominant breed types (Limousin, Charolais, Aberdeen Angus, Simmental, and Belgian Blue (86% of total)). We found in this high risk cohort that dairy breed animals tended to be significantly less likely to disclose with a VL, suggesting diagnostics for bTB may be behaving differently amongst herd types [25]. Recent research suggests that some tests may have differing characteristics depending on breed type [14], with data from chronic herds in Northern Ireland suggesting that the SICCT test may not perform as well in dairy herds as it does in beef herds [12]. Genetic susceptibility may also be a factor [62], with evidence from an Irish cattle population to suggest that Holstein cattle are the least susceptible breed examined, with Simmental and Charolais beef breeds being most susceptible [63]. In the current study dataset, for breeds with >300 animals represented, the highest proportion of animals confirmed were Charolais (46.08% confirmed bacteriologically) and Simmental (45.05% confirmed), while the least likely breeds to confirm were Friesian (32.22% confirmed) and Holstein (37.97% confirmed) at *post-mortem* (Additional file 4; also see [64]). It is currently difficult to ascertain whether the effect is due to increased resistance, non-specific skin test reactions or a reduced pathogenesis and lesion formation in dairy cattle [25]. However, there is evidence to suggest that confirmed breakdown recurrence is more frequent in dairy relative to non-dairy herds in Britain and Northern Ireland [65, 66]. Dairy herds tend to have an older age profile, which also affects risk (see below), however in this cohort there was little difference in the mean age of dairy breed animals (4.0 years) and non-dairy animals (3.8 years).

Age-class was another significant variable affecting the probability of an animal being confirmed (with VL; similar to [4]). The results of the present study suggested an increased risk for animals within the first and second quartiles, relative to other age-classes. Brooks-Pollock et al. [67] working in GB found that bTB risk increased with age up to the highest risk age group at 12 and 36 months old before risk declining. Potentially this pattern emerged due to the detection and removal of infected animals, with surviving cohorts of animals could have lower rates of infection. The same authors presented data on VL confirmation which suggested that confirmation risk declined with age. Anergy due to repeated testing could be a factor affecting the immunological response to the tuberculin tests with age [68, 69]. Interestingly, Clegg et al. [70] when undertaking a case-control study of non-reactor bTB confirmed animals in Ireland found that cases tended to be older than the national average age at slaughter, mirroring previous results [1] that suggested confirmation risk increased with age in non-reactors.

We found that the movement history of animals was a significant factor associated with increased probability of an animal being confirmed to have lesions found at abattoir. This suggests that movement through multiple premises may increase an animal’s risk significantly, or increase the pathological response if exposed during their lifetime. Lifetime moves were not previously associated with animal-level risk in Northern Ireland [4] in a study assessing the *post-mortem* confirmation risk of reactors. However, movement has been highlighted as a bTB risk factor in other studies (e.g. [71, 72]). In the present study, there was a significant positive relationship between increasing age and movement (*p* < 0.001), and with beef animals relative to dairy breed animals (*p* < 0.001). Therefore, the compounding effects of potential increased exposure (across multiple premises and animals), older animals, and being more likely a beef animal may have contributed to this finding. Stress due to movement may also be a significant driver of this pattern, as stress is known to negatively affect the immunocompetency of cattle [73, 74]. Lifetime number of herds an animal resided within significantly correlated with lifetime market moves and the number of animal purchases (i.e. buying in) the disclosing herds made in the 3 years before the disclosing test. Therefore, animals which resided in many herds, also made many market moves, and markets are known to be highly connected nodes within cattle trade networks in Northern Ireland [75] and elsewhere (e.g. GB, Denmark and Italy; [76–78]), which potentially increases exposure to infectious hosts and contributes to disease spread [70, 71]. Herds that purchased many animals also tended to buy-in animals with greater market-move histories (e.g. animals in herds in the fourth quartile of purchasing had on average 2.2 more market moves than animals in the first purchasing quartile; linear regression; *p* < 0.001). Finally, there was also an association between movement history and the time between the penultimate and disclosing tests – for example, for each additional market move, the time between tests increased by 5.5 days (linear regression; *p* < 0.001). While animals should be tested on average once per year in Northern Ireland (as part of an Annual Herd Test (AHT) or other follow-up tests), this may not always be the case with animals that move frequently across herds which undertake testing at different times of the year. While the average time between penultimate and disclosing test in this high-risk cohort was approximately 6 months (mean: 156 days), the longest time period between tests was almost 2 years (665 days). This pattern could be a factor in the time to detection of infection.

Finally, we provide evidence that the penultimate SICCT test result was significantly associated with *post-mortem* presence of a lesion at slaughter. Severe interpretation reactors were significantly more likely to confirm with a lesion at death. Similar results with a significantly increased risk of *post-mortem* confirmation in reactors has been demonstrated in other studies in Northern Ireland [4] and Ireland [70]. This could indicate that such animals in high-risk herds represent exposed animals in the early stages of infection that may have been missed by the SICCT test and so could pose a future risk [79, 80]. Indeed, Clegg et al. [79, 80] found that inconclusive reactors (animals which had reaction sizes of >2 mm on the bovine tuberculin injection site and between 1 and 4 mm > the avian response) had significantly increased future risk of bTB relative to non-reactor animals within the same herds or in comparison with animals in the national herds in Ireland. Furthermore, Lahuerta-Marin et al. [81] also showed how SICCT negative, but INF-γ test positive animals left on farm constituted to farms a significant future breakdown risk. In herds with significant recurrent problems, indicators of future risk could be considered when managing herd breakdown risk.

### Limitations and future direction

There were some limitations with regards this study. Firstly, post-mortem diagnostics for bovine TB has limitations in terms of variable sensitivity (e.g. see [12, 13]), though the slaughter house surveillance does exhibit high specificity (>99%). Similarly, abattoir carcass assessment for liver fluke infection can exhibit limited sensitivity and specificity at the animal level (e.g. 68%SE, 88% SP in Scotland; [82]), though there is no assessments of Northern Irish abattoir performance yet nor how transferrable the results are from other countries [29]. Part of the issue relates to inter-abattoir variation in surveillance effectiveness [1, 2], however, we largely controlled for this by having 99% of samples being processed through a single experienced slaughter house. While the sensitivity and specificity of the RT-PCR BVDV test is very high [>99%; 28], the animal level prevalence was very low (lower than expected given routine surveillance data), which may have diminished the power to detect an effect, if present. We did however, endeavour to overcome this, by assessing indirect effects of exposure, as BVDV is highly contagious within herds with actively excreting animals [28]. Future studies will directly assess the impact of MAP on bTB test diagnostics in Northern Ireland, to help refine and assess the validity of the present findings. Liver fluke exposure will be assessed systematically in Northern Ireland using a prospective study design assessing bulk milk antigens using high performance ELISA tests at herd levels, to assess the herd level nexus between liver fluke and bTB risk.

## Conclusion

There is significant animal-level variation in the *post-mortem* presence of VL from animals removed as part of bTB breakdowns in Northern Ireland. In this dataset, we found limited robust evidence of BVDV impacts on metrics of *post-mortem* confirmation, concurring with previous herd-level analyses. We did not find evidence to support liver fluke infection impacting on VL presence during this study. Exposure to *Mycobacteria* other than *M. bovis* may also have an impact on the SICCT test performance, with *M. avium* PPD reactors exhibiting higher probability of confirmation than non-reactor animals. We hypothesize that this effect could be partially due to exposure to *Mycobacterium bovis* subsp. *paratuberculosis* (MAP) the etiological agent of Johne’s disease, but this needs further confirmatory research. There was a strong significant relationship between visible lesion presence and increasing positive bovine tuberculin bias over the avian tuberculin reaction. Visible lesion presence in this high risk cohort was related to breed (decreased in dairy breeds relative to non-dairy), age (older animals are less likely to have lesions), movement history (increased risk with how many herds an animal resided in) and whether the animal was inconclusive to the SICCT at its penultimate test (previous test results can be a predictor of future risk).

## Additional files

Additional file 1: Figure S1. Graphical exploratory assessment of the relationship between the probability of bovine TB visible lesion (VL) presence and the bovine minus avian tuberculin (*b-a*) reaction sizes (mm) at the disclosing test for cattle culled in Northern Ireland during bTB breakdowns (*n* = 5698). The LOWESS curve (grey solid line) is a locally weighted regression line (bandwidth: 0.5); orange line represent *b-a* as a log-transformed predictor. Note, that the log-transformed predictor fails to address the increasing risk at negative *b-a* values, due to this the *b-a* variable was modelled as a categorical variable. (PDF 159 kb)

Additional file 2: Supplementary material 1. Detailed description of univariable associations between candidate predictors of TB-visible lesion presence. (PDF 333 kb)

Additional file 3: Figure S2. Relationship between the probability of an animal having a bTB visible lesion at slaughter and the time between the penultimate and disclosing skin tests in cattle in Northern Ireland. Orange line represents a locally weighted regression line (non-linear regression fit; LOWESS); Grey line represents the predicted fit from the log-transformed predictor variable (log(time in days)). (PDF 157 kb)

Additional file 4: Figure S3. The point estimated linear predictions from a random effects logit regression model for the presence of bTB visible lesions (VL) at slaughter amongst recorded cattle breeds for *Mycobacterium bovis* exposed cattle slaughtered in Northern Ireland. The grey dots represent point estimates; dots farther from zero line represent animals with higher or lower risk of disclosing with a lesion at slaughter relative to the population average. Orange circles represent weighted sample sizes. Outliers include (grey arrows): BGA = Belted Galloway highest risk of VL; SHB = Short horn beef cattle lowest risk of VL. Highly represented breeds are highlighted. (PDF 239 kb)

## Abbreviations

AUC: Area under the ROC curve
bTB: Bovine tuberculosis
BVDV: Bovine Viral Diarrhoea Virus
JD: Johne’s Disease
MAP: *Mycobacterium avium* subsp*. paratuberculosis*
VL: bTB visible lesion
