# Supplementary figures and images for: Bovine tuberculosis visible lesions in cattle culled during herd breakdowns: the effects of individual characteristics, trade movement and co-infection
Source: BMC Vet Res. 2017 Dec 29;13:400. doi: 10.1186/s12917-017-1321-z (PMC5747088; doi:10.1186/s12917-017-1321-z)

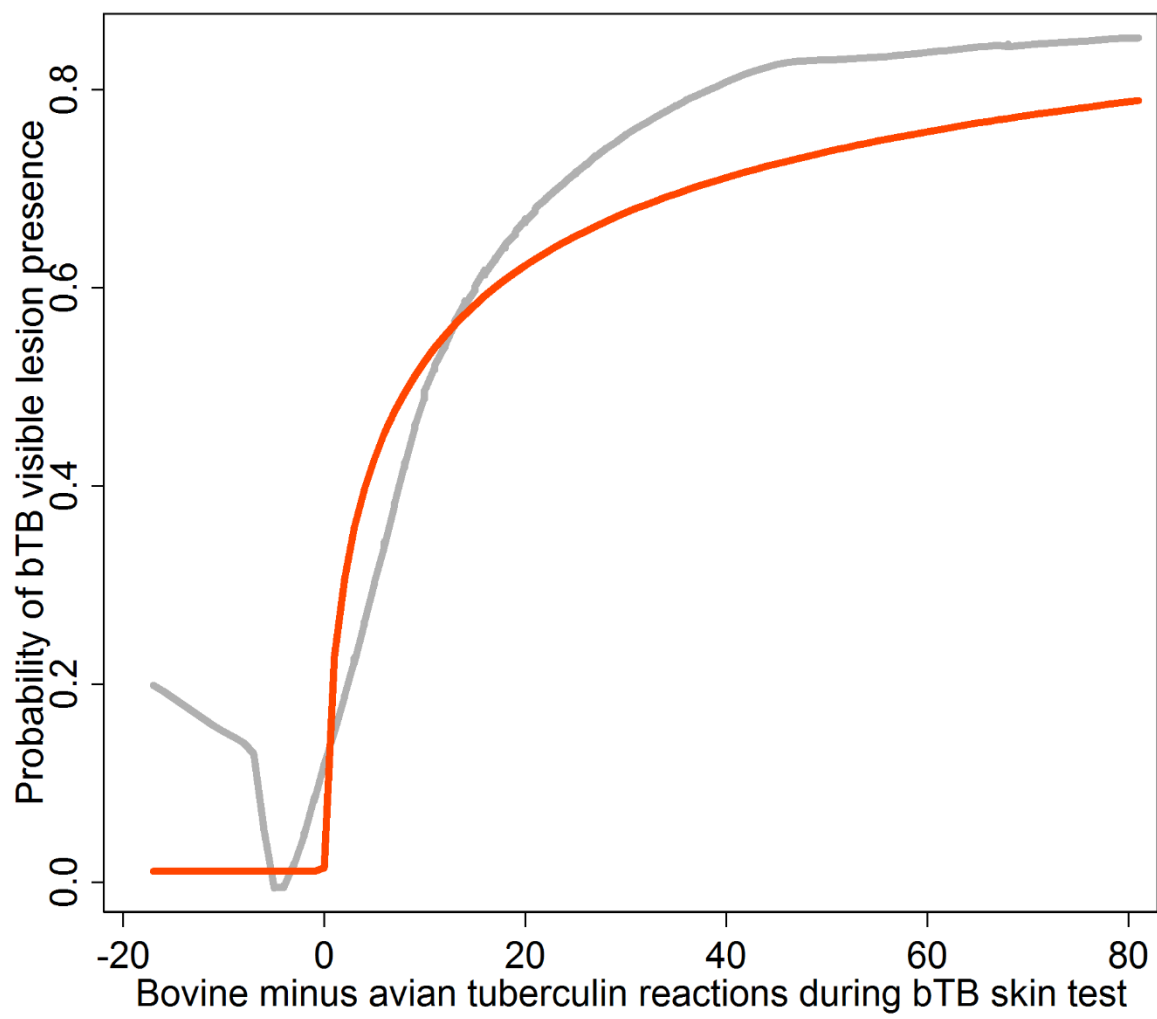

Supplement: Supplementary file 1 — Graphical exploratory assessment of the relationship between the probability of bovine TB visible lesion (VL) presence and the bovine minus avian tuberculin (b-a) reaction sizes (mm) at the disclosing test for cattle culled in Northern Ireland during bTB breakdowns (n = 5698). The LOWESS curve (grey solid line) is a locally weighted regression line (bandwidth: 0.5); orange line represent b-a as a log-transformed predictor. Note, that the log-transformed predictor fails to address the increasing risk at negative b-a values, due to this the b-a variable was modelled as a categorical variable. (PDF 159 kb) [file 12917_2017_1321_MOESM1_ESM.pdf]

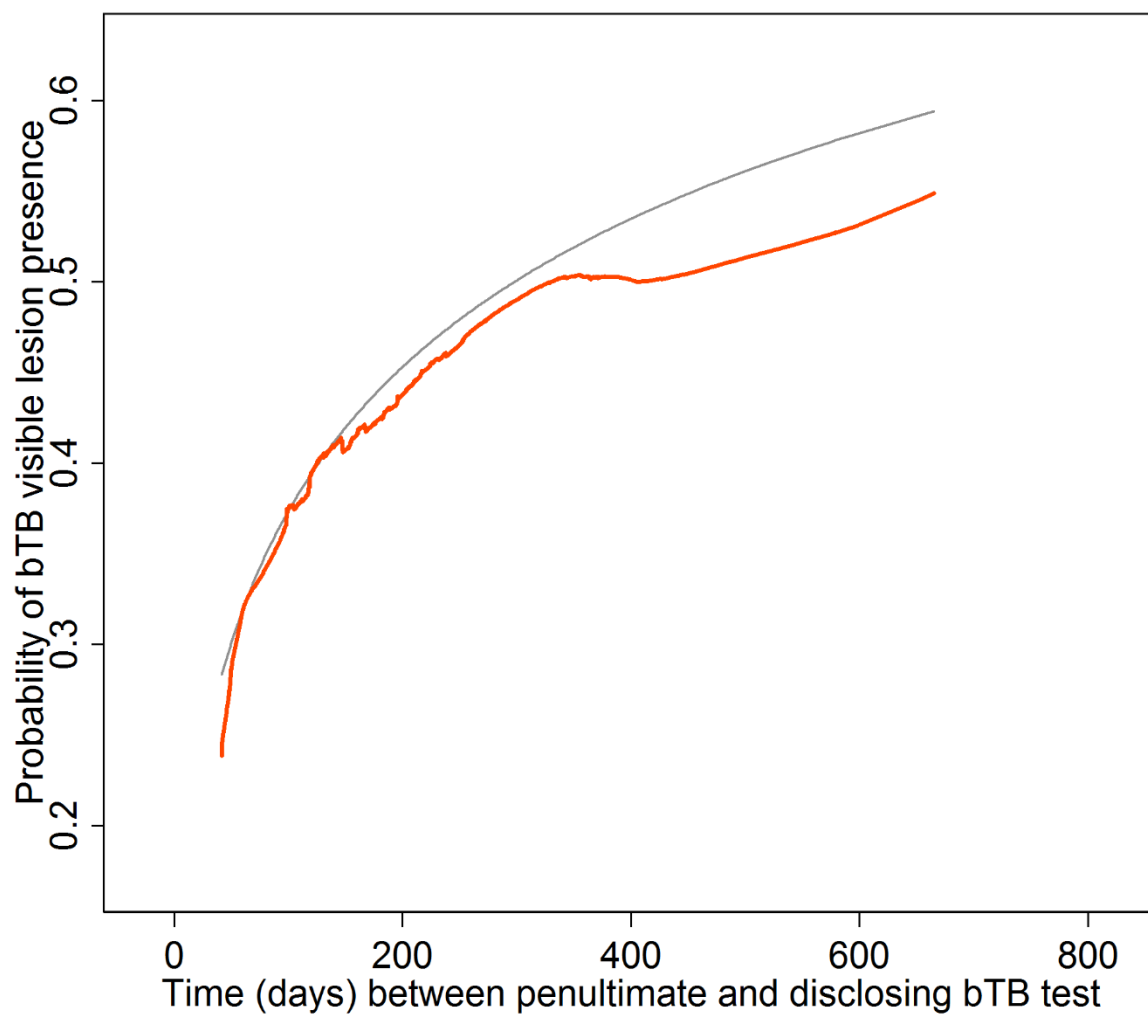

Supplement: Supplementary file 3 — Relationship between the probability of an animal having a bTB visible lesion at slaughter and the time between the penultimate and disclosing skin tests in cattle in Northern Ireland. Orange line represents a locally weighted regression line (non-linear regression fit; LOWESS); Grey line represents the predicted fit from the log-transformed predictor variable (log(time in days)). (PDF 157 kb) [file 12917_2017_1321_MOESM3_ESM.pdf]

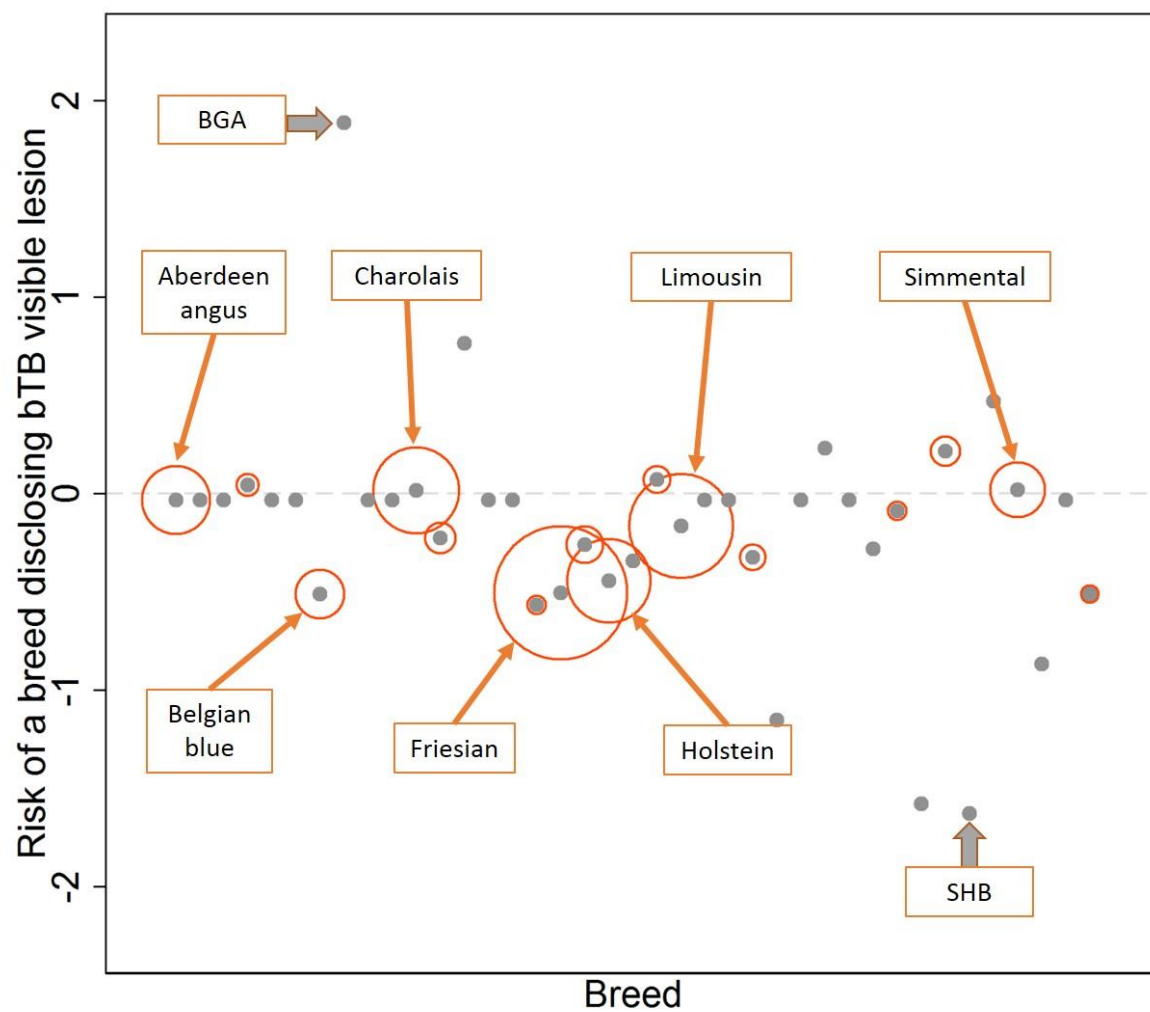

Supplement: Supplementary file 4 — The point estimated linear predictions from a random effects logit regression model for the presence of bTB visible lesions (VL) at slaughter amongst recorded cattle breeds for Mycobacterium bovis exposed cattle slaughtered in Northern Ireland. The grey dots represent point estimates; dots farther from zero line represent animals with higher or lower risk of disclosing with a lesion at slaughter relative to the population average. Orange circles represent weighted sample sizes. Outliers include (grey arrows): BGA = Belted Galloway highest risk of VL; SHB = Short horn beef cattle lowest risk of VL. Highly represented breeds are highlighted. (PDF 239 kb) [file 12917_2017_1321_MOESM4_ESM.pdf]
