# Supplementary material for: Bovine tuberculosis visible lesions in cattle culled during herd breakdowns: the effects of individual characteristics, trade movement and co-infection
Source: BMC Vet Res. 2017 Dec 29;13:400. doi: 10.1186/s12917-017-1321-z (PMC5747088; doi:10.1186/s12917-017-1321-z)
Supplement: Supplementary file 2 — Detailed description of univariable associations between candidate predictors of TB-visible lesion presence. (PDF 333 kb) [file 12917_2017_1321_MOESM2_ESM.pdf]

Supplementary material 1: Detailed description of univariable associations.

b-a PPD tuberculin reactions

There was a significant positive relationship between the probability of an animal displaying a visible lesion at slaughter and the *b-a* reading from the SICCT test. However, this relationship is non-linear (Supplementary figure 1). A LOWESS curve suggested that for positive *b-a* results, the relationship could be modelled as a logarithmic growth curve (achieved by transforming the *b-a* predictor). The logarithmic growth curve suggested a good fit, however the distribution ignored negative values – suggesting that the probability reaches 0 at *b-a*<0. The linear predictor overestimated the proportion with VL at slaughter when *b-a* is >30mm. This was verified when a categorical variable is used in the model. For every unit increase in log-*b-a*, the risk of an at-risk animal being disclosed with a VL increased by 2.02 (P<0.001).

As *b-a* can measure exposure to both *M. bovis* and *M. avium* exposure, we modelled the variable by categories (see main text).

Reason for removal

There was a significant relationship between the odds of VL and the reason for the animal's removal. Animals removed due to their SICCT test interpretation had an odds ratio of 20.83 relative to animals removed due to being negative-in-contacts (p<0.001). SICCT negative, IFN-γ positive, animals were non-significantly higher risk of VL relative to negative in contact animals (OR: 1.32; p=0.360).

BVDV

Overall, there were 6,242 animals with BVD test results of which only 17 were positive to the RT-PCR test (0.27%). The presence of BVDV positive animals in a herd indicates that the virus was circulating within the herd. There were a total of 286 direct or indirect positives within the dataset (4.6%). At a univariable level, there was no significant association between BVDV and lesion presence (OR: 0.33; p=0.106) or between indirect exposure and lesion presence (OR: 0.60; p=0.165).

Liver fluke

There were 6,242 observations in the dataset with *post-mortem* fluke infection status recorded. Of these, 361 (5.8%) were recorded with active fascioliasis. There were 876 (14%) animals with fluke damage (evidence of previous fluke infection in the liver). The overall level of fluke infection (either fascioliasis or fluke damage) in the dataset was 20% (1,236). Overall, none of the metrics of fluke infection were associated with the presence of bTB VL (p>0.3; table 3).

Age

The total number of animals with known age in this cohort was 6,231; missing animals mainly being from imported animals where birth date was unknown. Age ranged from 0.02 (8 days) to 18.67 (6814 days) years, with a mean age of 3.89 years (1418 days). There was significant variation in VL risk according to age. The relationship was non-linear (LOWESS plot not shown), and so was modelled using the quartiles of the age of animals (in a year scale). There was significantly higher risk of VL in animals in category 2 (from 1.53 to 2.85 years old) than any other category (2 v 1: OR: 0.77; P=0.005; 2 vs. 3: OR: 0.69; P<0.001; 2 V 4: OR: 0.76; P=0.004). However, there was no significant differences between categories 1, 3 or 4 (post-hoc Wald tests: p>0.2).

Breed

There were 39 breeds represented in the dataset. Each breed was represented on average 160 times, however the distribution was skewed with a few common breeds dominating (median = 10). Due to a lack of samples per type, a model including all breeds removed 12 breeds (remaining n=6225). Overall, the model did account for significant variation in risk (p=0.01). Belted Galloway (BGA) had the highest predicted probability of disclosing with a VL, whereas short-horned beef (SHB) cattle were least likely. However, the breeds with the most extreme values were associated with the smallest number of samples in our dataset (see supplementary figure 4 for weighted graph). Therefore, we reduced the breed diversity into a binary case of dairy and non-dairy breed types.

Overall, 2,817 animals were considered a dairy breed (45%). Dairy breeds were dominated by Friesian and Holstein breeds (91% of total). 35% of dairy breed animals (987/2817) had VL at slaughter. Non-dairy breeds were dominated by Limousin, Charolais, Aberdeen Angus, Simmental, and Belgian Blue (86% of total). 44% (1517/3425) of non-dairy breeds had lesions at slaughter. These differences were significant, the odds of a dairy animal having a lesion was 0.73 times that of a non-dairy animal ($p<0.001$).

Sex

Overall, 4,925 animals were female (79%). There was no significant difference between male and female cattle and their probability of disclosing with a VL (OR: 1.07; $p=0.406$).

Number of herds the animal was associated with from birth to death

The average animal within the dataset resided in 3.28 herds (SD: 2.45; Median: 2; IQR: 2-4) over its lifetime. The relationship between the presence of a VL and the number of herds was non-linear (assessed by LOWESS curve), and was modelled as a logarithm transformed predictor.

The risk of an animal having a VL at slaughter increased by 2.84 times for each unit increase in log-number of associated herds ($p<0.001$). All animals with only 1 associated herd had no VL at slaughter (n = 41). For animals with 2 associated herds, 37% had a visible lesion (n=1,357/3,697). In comparison, 72% (n=229/318) of animals which have resided in >7 herds had VL at slaughter.

Lifetime market movements

This variable counts the number of times an animal undertook a recorded “m” move within the APHIS dataset. This includes animals that move out to markets and back to the originating herds. The mean number of market moves made by animals in the dataset was 5.05 (SD 1.71; Median: 4; IQR: 4-6). There was a significant positive relationship between VL risk and market movements (OR: 1.04; $p=0.028$).

Time since penultimate test

There were 5,831 animals with a time between the penultimate and disclosing test. The animals with missing times were missing the date of penultimate test, the majority of which were imported animals. Also, first test animals were included in this cohort. There was a significant non-linear relationship between times since penultimate test and VL risk (LOWESS; see Supplementary figure 3). This relationship was modelled by taking the logarithm of time; the OR for each unit increase in log time (days) was 1.59 (p<0.001).

Herd history

The history of the herd in which the animal resided during the disclosing test was measured by assigning a 1 to herds with a breakdown in the last 4 years (excluding the current breakdown); a 0 if no breakdowns occurred. Overall, 2,926 resided in herds which had a breakdown in the previous 4 years (47%). Of these, 1,316 (45%) had a lesion, in comparison to 36% animals from clear herds had VL (N=1,188/3,316). There was a significant increased risk of an animal having a VL if they resided in a herd with a history of TB (OR: 2.13; p<0.001). There was an also increased risk of VL in animals which resided in herds with historic TB risk prior to their final herd (penultimate herd risk; OR: 2.02; p=0.044).

Penultimate SICCT reading

Animals which were penultimate severe positive reactors had 2.42 (p<0.001) times the risk of having a VL than inconclusive severe reactors. There was no significant difference between negative and severe inconclusive reactor animals (p=0.84).

Breakdown size

In total, there were 6,242 records with associated breakdown size. The mean breakdown size was 27.10 (SD: 36.45; IQT: 6-30); however, the distribution was highly skewed, therefore the median was much smaller at 14 animals. There was a negative relationship between breakdown size and VL presence, with an OR of 0.992 for each additional animal disclosed during a breakdown (p=0.001).

Herd size

The LOWESS curve suggests a non-linear relationship between VL risk and herd size. Herd-size was categorised into quartiles for model fitting. There was significant variation overall between the herd size quartiles and VL risk (p=0.044). There appeared significantly lower risk of VL in animals from herds in the 3rd (0.710; p=0.013) and 4th (0.722; p=0.019) quartile relative to the first quartile. However, there was no difference between quartile 1 and quartile 2 (p=0.240).

Purchases

There was a very large skew in the number of purchases between farms (mean: 270.81; median: 32; IQR: 8-101). There was no overall significant relationship between purchases and VL risk (p=0.114).

Region

DVO was modelled by categorising the regions into 3 areas following Lahuerta-Marin et al. (2015). Overall, there was no significant variation in VL risk between regions (p=0.171).
